# Malpractice*Read before the Buffalo Medical Association, Dec. 2d, 1879.

**Published:** 1880-01

**Authors:** George W. Clinton

**Affiliations:** Late Chief Justice of the Superior Court of the City of Buffalo


THE BUFFALO

MEDICAL AND SURGICAL JOURNAL.

VOL. XIX.—JANUARY, 1880.—No. 6.

### ORIGINAL COMMUNICATIONS.

### MALPRACTICE.*

BY GEORGE W. CLINTON, LL. D.,

Late Chief Justice of the Superior Court of the City of Buffalo.

*Gentlemen*:—Respect and esteem for my many friends in the Buffalo Medical Association led me to accept your cordial invitation to prepare for you a paper on medical malpractice. It will, I know, be an offering but ill-proportioned to my veneration for your profession and my desire to be of service to its practitioners in our city. To treat this great topic ably, one should be a sound lawyer, and a thoroughly educated physician and surgeon. Whatever I may be as a lawyer, my knowledge of your profession is naught. True it is that, a little more than half a century ago, I devoted two years of my life to the study of medicine and its auxiliary sciences, with reasonable facilities and under able teachers, and I hope that I will not be deemed immodest in declaring my belief that, if I had continued my studies another year, I should have graduated with honor. But, thank heaven, science and art are not and never have been stationary. The

* Read before the Buffalo Medical Association, Dec. 2d, 1879.

mind is never satisfied with its possessions, and the triumphs of art and acquisitions of knowledge, precious as they are in their fruition, are the strongest incentives to continued research and new effort. Hippocrates, like Æsculapius, is a famous but unfruitful name. The Hippocratic collection seems of interest only as material for the history of the art. The name of Galen is justly honored, but I apprehend that the utter destruction of all his writings would not deprive modern practitioners of a necessary or useful guide in any matter of sound practice, or blot out a now accepted theory. Probably neither of the three, were they now resuscitated and forbidden to add to their professional learning and implements, would be found fit for a place in this honorable body.

What another half century may do for the elevation of the profession, is a mere matter of conjecture; but, judging from the past, I augur great things for it. And here allow me to suggest, in passing, the possibility that, in therapeutics, the weight of the air and its hygrometric and electrical conditions may become, in many cases, matters to be taken into account. Certainly the past half century has been very fruitful of improvement. Sure am I that were I possessed of all the knowledge and all the skill I could have won in the two years I have alluded to and a third one added, and nothing more, I would not dare to present myself as a candidate for the degree of Doctor of Medicine. If I did, I am sure I would be kindly but peremptorily rejected. And so I know that I am not able to do full justice to the subject you have assigned me, and must again crave your kind indulgence.

In its most general sense, malpractice is illegal or immoral conduct. Webster also defines it as "practice contrary to established rules; especially professional misconduct of a physician." I am not satisfied with this definition. Professional misconduct of a physician is not always malpractice, although, if knowingly committed, it is ill-doing. The misconduct may be the fruit of innocent error. To be malpractice, in the technical sense, it must be the fruit of a want of due degree of skill and care, and subject the patient to appreciable and unnecessary injury. The lawyer, in relation to his client, occupies precisely the same relation that the physician or surgeon occupies in relation to his patient, and the duties and liabilities of the two are identical in definition and in measure. It is only, however, of medical malpractice that I shall speak. That, in my opinion, is such professional mistreatment of his patient as injures the patient and subjects the practitioner to a civil action for damages. This seems, at any rate, to be the ordinary understanding of the word. But, for the purposes of this paper, I will broaden the definition, so as to include instances of professional maltreatment and misconduct, which the law expressly denounces and punishes as crimes.

We might perhaps truly say that malpractice is the opposite of good practice; but this aids us no more than if we were to declare that good practice is the opposite of bad practice. So the definition I have given is simply a broad generalization, intended, like a great net drawn around a shoal of fish, to include them all. We may be sure that "all is fish that comes to our net!" but may still be ignorant of the essential structure and organization which constitute a fish and distinguish and separate it from all other animated orders. If we are not informed of these essentials, we may possibly call an eel a snake, and deem the whale a fish. What, then, are the essential qualities of malpractice? The general answer is, in every imaginable case of malpractice, that the practitioner has been guilty of a breach of duty to his patient. By assuming the case professionally, he, in the law, impliedly contracted to judge and treat it with a degree of professional care and skill, and has failed in doing so, to the injury of his patient. Men in every occupation vary in carefulness, in knowledge of and in ability in the occupation. As to care, which includes watchfulness, diligence, attendance and precautionary inquiry and examination when needed, it is generally regarded by lawyers as existing in three degrees, as is negligence its opposite. These degrees are thus distinguished in the books: ordinary care is that diligence which the generality of mankind use in their own affairs, and the want of it is ordinary negligence. That care which very inattentive and thoughtless persons never fail to take of their own affairs may be called slight care, and the want of it is gross neglect. The omission of that care which very attentive and vigilant persons take of their own affairs is slight neglect. Skill would seem to be regarded by the law as divisible into like degrees. It is possible that the law may recognize slight skill and gross unskillfulness, and great skill and slight unskillfulness, and the books and decisions do use, in reference to medical practice, the term ordinary skill; and they expressly declare that the surgeon and physician contract only for the exercise of ordinary care and skill, and are responsible only for the want of ordinary care and skill. *Omnis definitio in lege periculosa est.* These attempts at definition are not wholly satisfactory. They are indefinite and lack precision. Between slight and ordinary negligence on the one hand, and ordinary and gross negligence on the other, the boundaries are uncertain. Slight negligence and gross negligence, when presented before us, are recognized at once, but a case of ordinary negligence may seem slight to some, and gross to others. Occasionally a court has, apparently, attempted to define more specifically the meaning of ordinary care and skill, and used language liable to be so construed as to demand of the practitioners, in extraordinary cases, an extraordinary degree of, if not the highest, skill.

In Landon vs. Humphrey, 9 Conn. 242, a case of malpractice, the court said: "If there was either carelessness, or a want of ordinary diligence, care and skill," the plaintiff was entitled to recover the damages consequent therefrom. The words "either careless or" are either repugnant to "a want of ordinary diligence" and "care," or merely redundant, and should be stricken out. They would seem to imply that slight want of care was sufficient to make the practitioner liable for the consequent damages—a proposition utterly opposed to the whole current of authority.

In Braunberger vs. Cleis, decided in a District Court of Pennsylvania (13 Am. L. Journal, 587), the learned judge says: "The more difficult the duty or operation, the greater is the degree of care and skill requisite for its successful accomplishment; and in the performance of very difficult and dangerous operations in surgery, the surgical practitioner is required to possess and employ a higher degree of care and skill than would be necessary for the performance of operations less difficult and dangerous. But he is only required to employ a reasonable degree of care and skill in the operation, and in the previous and subsequent treatment of the case—that is to say, such a degree of care and skill as men of ordinary prudence, learning and skill in this department or profession"—(surgery, I suppose) "usually possess and employ, and if he does not, he is responsible for the injury occasioned by negligence or unskillfulness in this respect."

In Long vs. Morrison, 14 Indiana R. 505, this language is used: "A physician is liable for damages arising as well from the want, as from the want of application, of skill. It is his own fault if he undertakes without having sufficient skill, or if he applies less than the occasion requires." These propositions are, probably (I have not the reported case before me), qualified by the context. They would seem to lead to conclusions not sanctioned by the law.

In Iowa (Smothers vs. Hawks, 34 Iowa 86, and Almond vs. Ungent, 34 Iowa 300), ordinary medical skill and diligence is declared to be "the average of that possessed by the profession as a body, and not of the thoroughly educated only, having special regard to the improvement and advanced state of the profession at the time of the treatment."

In Patten vs. Wiggin (51 Maine 594), the court says of medical practitioners: "They are bound to use their best skill and judgment in determining the nature of the malady, the best mode of treatment, and in all respects to do their best to secure a perfect restoration."

In reference to the foregoing extracts from decisions, it is necessary to bear in mind that the opinion of the Court is to be read and construed in connection with the facts of the case decided. When we faithfully do this, we often find that a different and consistent interpretation plainly flows from language which we otherwise might have regarded as incongruous and unreasonable. So construed, I have found no reported case in which the doctrine is asserted or acted upon, that the medical and surgical practitioner is bound to the possession and exercise of a higher degree of care and skill than that which the law calls ordinary. I hardly know how to define that degree. It can hardly be the average of the care and skill of the profession. It is impossible to ascertain that average, and, if ascertained, it would be impossible to express it and mold it into a rule or standard. I should say that the degree of care and skill required by the law of the physician and surgeon, whether we call it ordinary or not, is that degree the exercise of which generally satisfies the consciences of physicians in good standing. Whether it has been exercised or not cannot be determined, in the absence of gross negligence or plain mispractice, without a dispassionate consideration of the whole case. A mistake in the diagnosis may lead to a wrong treatment, and result disastrously. But it by no means follows that the physician was professionally ignorant, or incautious in his conclusions as to the disease, or in the treatment consequent upon his conclusions.

In an action for malpractice, the defendant who, being well educated in the profession, in his treatment of the plaintiff, acted in accordance with his conscientious convictions of duty, may almost certainly rely upon acquittal. The failure to cure establishes no default on his part. Physicians of ordinary skill are far from adopting in similar cases the same medicines, appliances, and means of cure. There is room here for a wide diversity of practice. If he has not and cannot timeously procure the most approved medicaments, and the most perfect surgical instruments and implements, it argues neither carelessness nor a want of ordinary skill to use what, in his judgment, are the best substitutes he has at hand. In the excusable absence of quinine he may use Fowler's solution. If there be no tourniquet at hand, he will have recourse to the compress and the handkerchief to close a cut artery. I am not prepared to say that under imaginable circumstances he would not be justifiable in cutting off a broken and mangled limb with a jack-knife, and thrusting the stump into melted pitch.

Whether it be prudent or imprudent in a young surgeon who has had but little practice in his profession, and few or no opportunities to witness difficult operations, to undertake a difficult and precarious one, is altogether dependent upon circumstances. They may imperatively command him to attempt it. Life, or consequences as deplorable as death, may depend upon his declension to proceed at once. In such a case his conscience is clear, and there can be no legal liability for the result. If, however, there be time to call in an experienced surgeon, and he rashly takes sole charge of the case, and performs the operations carelessly and unskillfully, he must abide the condemnation of the law. A physician or surgeon is not absolutely bound by the diagnosis of others superior to him in reputation, but may act in accordance with his own. I read in a newspaper, many years ago, a statement of which this is a summary. Eminent surgeons examined a patient, and determined that he was suffering from a deep-seated aneurism. A young surgeon (a name was given) concluded that it was an abscess, took charge of the case and used the bistoury; pus followed, and the patient was restored to health. A subsequent similar case occurred, and his diagnosis was the same, but he was mistaken, and the bistoury caused immediate death, whereupon he went home and committed suicide. This dreadful result proves nothing. A properly careful diagnosis, though mistaken, justified the treatment.

With the death of the surgeon or physician a right of action against him for malpractice absolutely abates; and the action in like manner abates upon the death of the wronged patient. One who is injured by the wrongful violence or negligence of another has an action for the damages sustained by him, but, if his death be caused by the violence or negligence, his right of action abates, and the common law gives no remedy to his personal representatives, or widow, or next of kin. In 1847, a law was enacted by our Legislature, giving an action to the personal representatives of any person whose death should be caused by the wrongful act, neglect, or default of another, in case such person, if he had survived, would have had an action for his damages against the wrong-doer. In this action the jury may give such damages (not exceeding $5000) as they shall deem a fair and just compensation with reference to the pecuniary loss resulting from the death to the widow and next of kin, among whom the recovery is to be distributed as in cases of intestacy. It is to be observed that the statute expressly provides that the wrong-doer shall be liable to this action, "notwithstanding the death shall have been caused under such circumstances as amount in the law to felony"—that is to say, to a crime punishable by imprisonment in a State Prison or by death. This provision abrogates the common law doctrine that in felony injurious to a private person, the private injury is merged in the crime so that, at law, the personal injury is irremediable.

That where malpractice causes death, the surgeon or physician is liable to this statutory action, there can be no reasonable doubt. In Braunberger vs. Cleis, before cited, the practitioner was held to be liable, under a similar statute, giving an action for every death caused by illegal violence or negligence. It is very clear that a death caused by malpractice is caused "by a wrongful act, neglect or default," within the meaning of our statute. Indeed the case of Quin vs. Moore, which originated in our city, and is, I presume, fresh in the memory of some of you, is decisive of the question. An apothecary's clerk sold a boy's mother morphine for quinine. It was duly administered to the boy as quinine, and caused his death, and the Court of Appeals, our highest court, held that the apothecary was liable to damages for the death under the statute.

The physician must be a hero. He must expose himself to dangers more imminent than those of battle. He must accept cases of infectious and malignant disease. But he is bound in conscience and by law to use every reasonable precaution against his conveying it to others. In Kentucky (12 B. Mon. 467) a physician who, while attending a person laboring under such a disease, neglected to take the usual precautionary measures to purify himself, and consequently communicated it to another patient, was held subject to the latter's action for damages.

A physician or surgeon may, of course, at the instigation of the devil, intentionally kill his patient by poison or other maltreatment. In such case he is, of course, guilty of murder, and would be liable to an action under the statute I have referred to.

So, under the like influence, he may prescribe medicine for a pregnant woman, or use, or advise her to use, mechanical or other means of procuring abortion, with intent to produce a miscarriage, where such miscarriage is not necessary to preserve the life of the woman. In such a case, if a miscarriage does not take place, or does occur and is not fatal, either to the mother or the child, the physician (or other person) is guilty of a felony, punishable by imprisonment in a State Prison for not less than one, nor more than three years. In this case, you will observe that the mere prescription or advice with the intent to produce a miscarriage, constitutes the crime. But if the prescription, or use of the medicine, or the use of the mechanical means, with the intent to produce a miscarriage, result in the death of the mother or the child, then the physician (or the person) is guilty of a felony punishable by an imprisonment of not less than four nor more than twenty years.

Our law recognizes the duty of the physician to keep, as far as possible, his mind clear and strong, and his powers of observation unimpaired, when dealing with his patients. This is manifest in this statutory provision: "If any physician, while in a state of intoxication, shall, without a design to effect death, administer any poison, drug or medicine, or do any other act to another person" (patient) "which shall produce the death of such other, he shall be deemed guilty of manslaughter in the third degree." That crime is punishable by imprisonment in the State Prison for not less than two, nor more than four years.

Our Revised Statutes, after defining murder, justifiable homicide, and three degrees of manslaughter, add the following enactment: "Any other killing of a human being by the act, procurement, or *culpable negligence* of another, when such killing is not justifiable or excusable, or is not declared in this chapter murder, or in this title manslaughter, of some other degree, shall be deemed manslaughter in the fourth degree." It seems to me that if the death of a patient be produced, proximately, by the neglect of the physician, so as to render the physician liable for damages to the widow and next of kin, under the act of 1847 above referred to, he is also guilty of this crime.

I might properly—perhaps it would be better to—end this paper here. But, my friends, my regard for you impels me to remark upon another theme which is alien to the topic you assigned to me. I refer to your duty and deportment as witnesses in courts of law. You are liable to be called upon, and are frequently called upon, to testify, as experts or otherwise, and it seems to me, I am free to say, unjust that, in the matter of compensation, you should not be distinguished from ordinary witnesses. As witnesses, you are compelled to submit to pecuniary loss, and too often to put the health and safety of your patients in hazard. The testimony of physicians, surgeons and chemists, as experts, are often indispensable to the administration of justice. When true and properly delivered, it is a sure shield of innocence and detection of guilt.

At common law, the disclosures of the patient to his physician are not sacred; and the physician, as a witness, is compelled to testify as to the communications of the patient, however necessary, to enable him to judge and prescribe. The Revised Statutes contain an enactment, which, as re-enacted by the code with some change of phraseology, reads thus: "A person duly authorized to practice physic or surgery shall not be allowed to disclose any information which he has acquired in attending a patient, in a professional capacity, and which was necessary to enable him to act in that capacity" (Code, § 832). This language is extremely broad and comprehensive. My belief is that the courts will construe it as applying only to information derived from the patient, and apply it only to actions and indictments in which he is directly interested. Surely, if a man be injured by another, the physician who attended him must be admitted, on the trial of the injurer, to prove all of the injured man's disclosures, and his bodily condition and symptoms. In Johnson vs. Johnson (4 Paige, 460), Chancellor Walworth held that the testimony of the physician was admissible, he only objecting. But, upon the reversal of that case in the Court for the Correction of Errors, Chief Justice Savage maintained, what is apparently the true doctrine, that the secrecy of the physician is solely the patient's privilege, and that the physician cannot testify as to the facts within the Statute prohibition unless the patient grants permission. A violation of the prohibition of this Statute is a misdemeanor (2 R. S., 696, § 39), and is punishable by imprisonment in jail, not exceeding one year, and by a fine not exceeding two hundred and fifty dollars, or by both such imprisonment and fine.

As to the delivery of testimony by you as experts, I have very little to say that might not just as properly be said to a witness who is called to testify only as to the facts of the case. The difference rests in this: The expert as such is asked only for his opinion upon the facts. He may be asked his opinion upon a hypothetical state of facts, and required to give reasons for the opinion he expresses. The cross-examiner is allowed great latitude, and, I am sorry to say, not infrequently abuses it. But if the witness will only remember the worth and dignity of his profession, and that he is there simply to speak truth, as a servitor of justice, no arts or sneers of counsel can disturb him. Calm and self-possessed, he will answer every question, direct or cross, fully, and in the plainest and most lucid language in which the meaning of the answer can be conveyed to the jury. To such an answer he will add nothing, unless it be a necessary explanation. He will not air his learning before the court, nor have any the least contention with counsel. The court will, if need be, protect him from the abuse of lawyers. Such a witness will retire from the stand as calmly as he went upon it, approved by his own conscience, and respected by the court, the jury and the bar.

